# Outdoor Navigation Assistive System Based on Robust and Real-Time Visual–Auditory Substitution Approach

**DOI:** 10.3390/s24010166

**Published:** 2023-12-27

**Authors:** Florian Scalvini, Camille Bordeau, Maxime Ambard, Cyrille Migniot, Julien Dubois

**Affiliations:** 1 Laboratory ImViA EA 7535, Université de Bourgogne, 21078 Dijon, France; cyrille.migniot@u-bourgogne.fr (C.M.); julien.dubois@u-bourgogne.fr (J.D.); 2 LEAD, CNRS UMR 5022, Université de Bourgogne, 21078 Dijon, France; camille.bordeau@u-bourgogne.fr (C.B.); maxime.ambard@u-bourgogne.fr (M.A.)

**Keywords:** navigation aid, wearable assistive device, obstacle avoidance, sensory substitution, visual impairment, deep learning

## Abstract

Blindness affects millions of people worldwide, leading to difficulties in daily travel and a loss of independence due to a lack of spatial information. This article proposes a new navigation aid to help people with severe blindness reach their destination. Blind people are guided by a short 3D spatialised sound that indicates the target point to follow. This sound is combined with other sonified information on potential obstacles in the vicinity. The proposed system is based on inertial sensors, GPS data, and the cartographic knowledge of pedestrian paths to define the trajectory. In addition, visual clues are used to refine the trajectory with ground floor information and obstacle information using a camera to provide 3D spatial information. The proposed method is based on a deep learning approach. The different neural networks used in this approach are evaluated on datasets that regroup navigations from pedestrians’ point-of-view. This method achieves low latency and real-time processing without relying on remote connections, instead using a low-power embedded GPU target and a multithreaded approach for video processing, sound generation, and acquisition. This system could significantly improve the quality of life and autonomy of blind people, allowing them to reliably and efficiently navigate in their environment.

## 1. Introduction

The World Health Organization’s (WHO) Vision 2020 initiative has estimated that there are over 43.3 million visually impaired people in the world, 36 million of whom are considered blind. The number of visually impaired people is projected to rise over the next few decades, reaching around 61 million in 2050, with the majority of them in developed countries [1]. Indeed, the main causes for this phenomenon include age-related macular degeneration and cataracts due to an ageing population, although diabetes and other non-communicable diseases also contribute to this increase. Although existing treatments such as surgery, lasers, or eye drops for glaucoma are available to halt and limit the progression of the disease, early detection is necessary. However, access to detection and treatment resources is limited in low-income countries, amplifying the risk of becoming totally or partially blind. Medical access to the necessary human and technological resources is crucial for preventing the progression of diseases, but improving the lives of people who are already blind is also a significant challenge. Indeed, current medical knowledge does not allow us to completely remedy the loss of visual perception in all blind persons.

In humans, vision dominates the other senses in tasks involving spatial information processing. Indeed, children use vision to calibrate their other sensory modalities [2]. Navigation in a dark environment is a good example of the impact of visual perception compared to the other senses, where humans prefer to navigate in places already seen in the first place without relying more on other senses such as touch, sound, and smell to understand their surroundings [3]. However, a lack of visual information due to total or partial blindness prevents the ability to access the visual memories of the environment and forces blind people to rely on their other sensory modalities. In this situation, brain plasticity can enhance human perception by rearranging neural pathways. However, neural plasticity decreases with age and, therefore, is not really effective for late blindness [4]. Moreover, sensory compensation is intrinsically limited by the detection threshold of the other senses [5].

The lack of spatial information due to severe blindness causes many difficulties in everyday life, especially during navigation. Moreover, our environment often includes written messages or pictograms that are not accessible to blind people. For these reasons, walking in an unknown environment remains dangerous. But the dangers differ between indoor and outdoor situations. The indoor environment is often a dense configuration with many static or slow-moving objects. In contrast, outdoor environments are sparser but with objects that can move quicker, especially dangerous areas such as crossways and roads. These mobility problems significantly decrease their quality of life by limiting access to social activities. This contributes to a feeling of being isolated with poor autonomy [6].

Throughout history, various methods and solutions have been developed to improve the autonomy of visually impaired people and facilitate their insertion into society. The most used remediation means include the white cane, the guide dog, and Braille writing. Guide dogs are especially useful for navigation assistance. However, their training requires a long period and a big investment, often running into tens of thousands of dollars. The white cane only provides information about obstacles in very close proximity. Guide dogs are mobility aids that help visually impaired people to find their way around, spot obstacles, and improve their sense of security and independence. However, the lengthy training period and high costs, often running into tens of thousands of dollars, are barriers for many people [7]. In addition, these aids have limitations in terms of the range of obstacle detection and the variety of information perceived.

Recently, assistive technologies have been more and more used by the blind community and a recent study proposed that assistive technologies can be classified into three distinct categories [8] namely visual enhancements [9], visual replacement [10], and visual substitution. A visual enhancement device improves the existing visual information, and therefore, excludes people with complete blindness from its scope. A visual replacement device directly transmits data to the visual cortex using an invasive system. Visual sensory substitution devices add new information to a benefited sensory modality to compensate for the lack of vision. Although all senses can be used for VSS, hearing and touch are primarily used. Visual-to-tactile substitution devices, and roads’ visual information into haptic feedback are transmitted through the user’s skin as a tactile vibration corresponding to the visual event. In contrast, thevisual auditory substitution device (VASD) replaces tactile feedback with auditory information to the user, such as The Vibe. This approach has been proposed to guide a user by emitting spatial sounds and tested in a car park [11]. The design of sensory substitution devices aiming to assist blind individuals in unfamiliar environments can be categorised into three distinct groups: electronic travel assistance (ETA), electronic orientation assistance (EOA) [12], and position location devices (PLDs) [13]. ETA focuses on the detection of surrounding obstacles, EOA guides the user to a desired destination, and PLD describes a current position. A realistic navigational aid for blind must address all these challenges simultaneously to enable safe navigation in an unfamiliar environment. Indeed, successful navigation involves identifying the user’s position and providing instructions for reaching the desired destination while avoiding obstacles.

This paper introduces an innovative system designed to facilitate the navigation of blind users in urban environments. This system addresses the notable absence of suitable devices for outdoor navigation for those with complete visual impairment, addressing the challenge posed by the absence of continuous human assistance. The user is guided by specialised sounds indicating the orientation required to follow the optimised trajectory to reach a destination. The trajectory is defined using spatial sensors (global positioning system antenna GPS; inertial measurement unit IMU) and a recorded map of the urban navigation area. The crude pathway information is corrected in real-time using RGBD information acquired from a camera to avoid obstacles and dangerous areas such as roads. The ground estimation and obstacle detection were carried out using robust and real-time deep learning techniques, ensuring minimum latency between data acquisition sound updates. The software uses a multithreaded approach to GPU optimisation to run on an embedded Nvidia Jetson Orin board. This optimised configuration ensures low power consumption and a lightweight system, making it ideal for real-world scenarios. Our offline method avoids cloud computing techniques and eliminates transmission latencies and security issues in areas with limited connectivity.

## 2. Related Works

In this literature review, we will focus on sensory substitution systems through the different methods of localisation (PLD), orientation (ETA), and obstacle avoidance (EOA), as well as the interaction interface, which are all essential elements for an effective system. The user’s location is a critical factor in the development of any navigation system, particularly for those who are severely visually impaired. An inaccurate method could provide incorrect information and mislead the blind person with the wrong information and direction. Currently, most navigational aids locate users through either visual or wireless signals. However, these methods differ in their limitations based on the environment in which they are used. Indoor environments are manipulable spaces that can be modified to solve the localisation problem. This can be achieved by inserting active or passive RFID tags [14], Bluetooth [15,16] antennas for triangulation, or by locating a unique visual identifier through a visual marker uniformly distributed throughout a building [17,18,19]. However, implementing these technologies may require the integration of new transmitter beacons or visual patterns, and can be costly to cover larger environments such as urban areas. Additionally, the signal strength can be weakened by obstacles such as walls or floors, requiring more transmitters for compensation. In outdoor environments, similar localisation technologies are used with visual or wireless methods that allow wide area coverage. Some VSSDs have used visual information to determine the current location of blind people by mapping the visual data into a known 3D environment with the visual SLAM algorithm [20,21]. Unlike other methods, visual approaches do not require an additional sensor external to the camera but require prior recording in an urban environment. Furthermore, to reflect changes in the urban landscape, the reference environment needs to be updated on a regular basis. Wireless systems are based on technologies such as GPS [22,23] or cellular technologies using trilateration on a satellite network or triangulation with nearby cell towers on the strength of signals received to determine the person’s position. However, GPS technology’s accuracy is limited to within 3 m, which may be insufficient for some systems, but this can be improved with dual GPS antennas. A method [24] proposes to combine GPS data with the detection of visual landmarks (tree, pole, etc.) with known positions to improve the position accuracy.

The person’s location, combined with a 2D or 3D cartographic knowledge of the navigation zone, enables an optimal path to be established depending on the distance or safety of the route. Many navigation systems are based on pedestrian or driver navigation services such as Apple Map, Google Map [23], or open source services [25,26]. Other methods apply an orientation algorithm such as Dijkstra [27] on a schematic graphical representation of the navigation space composed of nodes and edges, respectively, symbolising intersections and roads in an urban environment. The optimal path for a blind person can be determined by using a weighted edge based on different criteria, such as the distance of the road or the accessibility of the path [26]. However, using online services requires a constant remote connection and imposes a constant cellular connection, while the orientation algorithm could be time- and memory-consuming, especially on large graphs.

The defined path cannot be optimal for a blind person if it only relies on predefined information from a planar representation of the environment and does not consider obstacles or the spatial structure of the area. Indeed, the ideal path would be dynamic and adjusted in real time based on obstacles, such as a pole or bicycle, that are encountered along the way. Ultrasonic sensors are proposed to detect the presence and position of relevant obstacles like a sonar [28]. The number of sensors and their position at different heights can provide better knowledge of the environment and potential obstacles at the head level. Visual information of the environment is also used to detect and localise nearby obstacles by applying deep-learning methods [25]. Recently, the improved hardware performance coupled with an efficient deep learning architecture designed for mobile implementation [29,30] allowed for real-time execution with low power consumption. In addition, the semantic information of an object such as this nature improves the understanding of the degree of danger that this object represents. Object tracking is associated with detection during navigation, providing a system with information on the speed and direction of detected objects. This requires pairing detections of the same elements in a sequence of images. One SSD method proposes to track dangerous features in a scene and prioritise them according to their dangerousness using an assignment method based on Hungarian’s algorithm [31]. The navigation area can also be dangerous for a blind person, especially when crossing roads or near traffic. Roads not designated for pedestrians may be used by fast-moving vehicles such as cars, trucks, and bicycles. Pedestrians must stay in designated areas, such as pedestrian streets or pavements alongside the road, to navigate safely. This problem echoes the problems of autonomous vehicle navigation, where semantic segmentation methods are used to extract the areas accessible or not to vehicles by deep-learning [32]. By extension, these methods have been adapted by dedicated pedestrian navigation systems for blind people to segment pedestrian areas from dangerous areas [33].

Navigation and obstacle avoidance heavily depends on the accurate and timely delivery of navigation guidance and information to users. This information must be accessible for blind people to understand, accurate, and fast enough to avoid nearby hazards. EOA tactile feedback uses multiple vibration positions that symbolise an orientation for the user to follow the path. The motor position can be located on the different parts of the user’s body. A tactile-to-shoe system [25] proposes to guide users by foot stimulation with four vibrators corresponding to the front, right, left, and back directions. Similarly, other methods propose a belt of vibration motors. Some systems emit a vibration event corresponding to a nearby obstacle’s position, with different intensity levels according to its distance. VASD proposes to exploit the vast possibility of encoding to interpret the surrounding environment by the emission of verbal of the orientation [34,35] or nearby obstacle [36]. A verbal expression describes the urban scene with semantic information and is easily accessible to the blind person. Others exploit the structure of the sound emission, such as the frequency [37], the stereophonic [38] or the intensity [39] to create short spatialised sound by a specific encoding scheme. The emission of short sounds enables the quick emission of a sound to alert a close danger of the user. Furthermore, blind individuals can perceive both the dynamic and static attributes of their environment with remarkable precision [40]. The information encapsulated in the sound information could integrate additional semantics to the localisation, such as the colour in the SeeColor method or information useful for the navigation. Indeed, an indoor navigation aid system extends the localisation information with the nature of the surroundings or to distinguish the danger signal and the path information [17]. The navigation aid systems faced multiple challenges to be beneficial and not hinder daily life. Indeed, these systems must consider factors such as ergonomics, the robustness of the system, and the user acceptance of the substitution protocol [41]. Robustness is particularly crucial for these systems. In addition, the system must be designed to be independent of third-party equipment, such as remote servers, that may introduce variable transmission delays and distant connection errors due to low-connection speed or white spot. This independence is necessary to ensure that the system provides accurate and reliable feedback to the user. User-friendliness is also essential for effective sensory, auditory, or tactile substitution systems. The device must be portable and convenient for long navigation periods. Additionally, the system must emit signals discontinuously, only during critical events, to prevent overloading blind people’s cognitive systems unnecessarily. The approach proposed in the global system without remote connection is designed on an embedded board to limit power consumption. Our auditory signals are emitted when the user is confronted with a danger or to indicate the trajectory at a frequency adapted to the walking speed.

## 3. Materials and Methods

**Figure 1:**
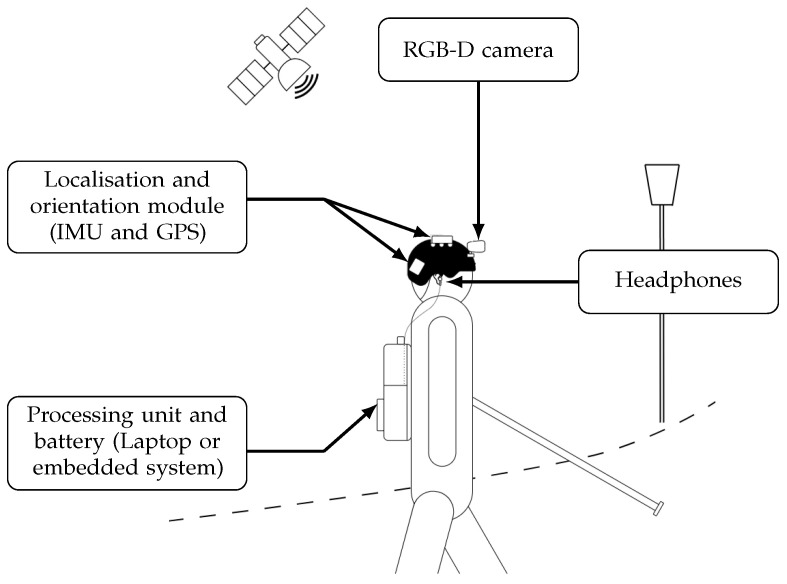
Schematic view of our navigation aid system consisting of a location and orientation module, an RGB-D camera, a processing unit, and a headset for sound emission.

We present a new assistive visual substitution system to aid individuals with visual impairments in their mobility within unfamiliar urban environments. The system offers auditory guidance, allowing blind individuals to navigate independently without external assistance or remote servers, ultimately enhancing their quality of life. The path information, essential in the navigation assistance, is provided through spatialised sound, delivering precise path information that incorporates the environmental topography of pedestrian routes. Simultaneously, a second sound cue indicates the presence of nearby obstacles, enhancing the user’s safety and situational awareness. Figure 1 provides a schematic view of our navigation system, highlighting the placement of components like the inertial measurement unit (IMU), GPS, RGB-D camera, and processing unit for user localisation and navigation information processing. The navigation system for blind individuals in urban areas relies on a series of recognisable landmarks. The user’s current location is used to determine a path, guiding them from one landmark to the next until they reach their intended destination. This spatial information is refined by visual clues to enable a safe way on a pedestrian way and to detect possible obstacles to ensure a safe navigation experience. The integration of trajectory information and obstacle alerts into a single, coherent auditory experience is achieved through advanced sonification techniques, enhancing the user’s awareness and understanding of their surroundings and providing clear directional guidance.

### 3.1. Localisation and Navigation

The position and orientation of a blind person when navigating in an urban environment are essential to providing the user with an appropriate path to follow and ensuring that they are travelling in the correct direction. Our system’s information is based on two key components: a GPS antenna and an IMU sensor. The IMU sensor provides information about the user’s acceleration, velocity, and head orientation in space. This is achieved through the fusion of data captured by different sensors, including a gyroscope, an accelerometer, and a magnetometer for 9 degrees of freedom IMU. The GPS antenna, on the other hand, provides the current geographical position of the user, including their latitude and longitude. This information is obtained by the trilateration of receiving signals from multiple GPS satellites in orbit around the Earth. Geographical location determines the starting point of navigation when the user wants to move around an urban environment.

**Figure 2:**
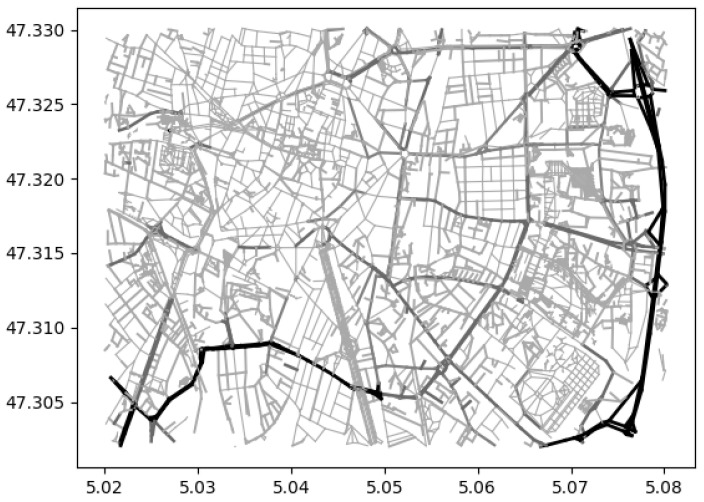
Representation of a 2D map showing the dangerousness of different roads in a city according to the road type or the presence of a sidewalk, with the lighter shades of grey indicating the low-danger roads and the black ones the most dangerous.

The geographical position of the initial user is combined with planar knowledge of the spatial structure of the urban environment. This map information allows the user to know the layout of the streets and their connections to estimate the most efficient route to a given destination directly and safely. The map information used in the navigation system is obtained from the open source mapping service OpenStreetMap, which provides an up-to-date 2D map of a given area and various information about its topology, including the presence of various features such as roads and buildings. The map provided is associated with attribute keys that provide detailed information about each feature, such as its name, type, and connections to other features. These data allow a better understanding of the environment and a focus on information and locations relevant to a pedestrian’s navigation. Their integration eliminates uncertain routes that lack meta-information or are too dangerous for pedestrians. These include major roads such as motorways and primary roads, which are intended for vehicular traffic and unsafe for pedestrians. Although the OpenStreetMap database relies on contributors and volunteers and may incorporate some labelling errors, most errors are commonly found on less-travelled paths, such as private roads or footpaths. These inaccuracies rarely pose a substantial threat to pedestrian safety due to the nature of the path and the incorporation of visual information to rectify the trajectory. The 2D map view in Figure 2 displays semantic information about the estimated level of danger on the roads for blind people in the city, with the latitude and longitude of the area on the axis. The estimated danger is based on the extraction of road data, specifically on the maximum speed, the type of road, and the presence or absence of information about pavements. Black areas are forbidden to blind persons, while light grey roads are more suitable for navigation.

Inaccessible areas such as black street are filtered out, along with building structure information and meta-information included in the file, to significantly reduce map file size and, indirectly, the route calculation time due to less information to process. The map file is filtered by an OpenStreetMap’s tool called *Osmosis*, according to the type of road or presence of pavement by maintaining the following attributes:

- Highway—road type: secondary, tertiary, pedestrian, service, residential, footway, steps, bridleway, path.
- Footway: sidewalk, crossing.
- Sidewalk: left, right, both.

**Figure 3:**
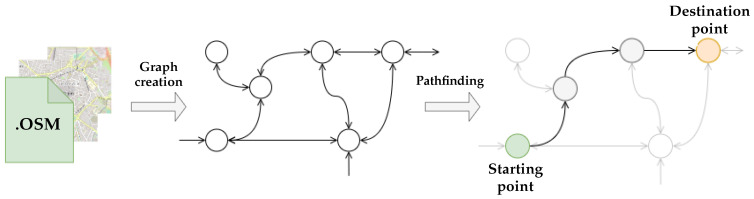
The diagram of the transformation of an OpenStreetMap into a graphical representation following a path to a specific destination via intermediate vertices.

The filtered map is transformed into a graph representation of the urban space with vertices and nodes. This graph’s vertices represent relevant geographical positions, such as the curvature of the roads and the presence of intersections. The edges represent the links between two vertices (Figure 3). The initial geographical position of the user, obtained through GPS, is used in conjunction with the structure of the urban environment to determine the starting point of the navigation path. The geographical position of the severely visually impaired person is mapped to the nearest vertex in the graphical representation to define the starting point of the graph. However, traversing all the graph nodes to find the closest vertex can be computationally time-consuming, especially in a large region with many vertices. Therefore, the graph is divided into sub-areas and gridded to limit the search space to a neighbourhood of a few hundred metres instead of a large region of several tens of kilometres. This gridding process allows the navigation system to considerably reduce the time needed to search for the vertex, avoiding waiting times without any disturbing information, which can lead to feelings of isolation.

The path navigation is performed on the graph representation with a way-finding algorithm to find the adapted path between the actual position and the desired destination. This predefined path must compromise between a short and a safe path to obtain a customised path for a blind person. Indeed, a short path can be composed of dangerous elements and make the navigation complicated. In contrast, a longer but safer path will increase the time of use of the cognitive abilities of the blind person. In order to reflect this problem of finding the adapted path on the graph representation of the environment, a weighting of the edges of the graph is performed based on the distance between two vertices and the complexity of the road using an empirical accessibility score (Figure 2). This score is mainly determined by the presence of pavement. Without information on pavements, the type of road and the speed limit are considered.

**Figure 4:**
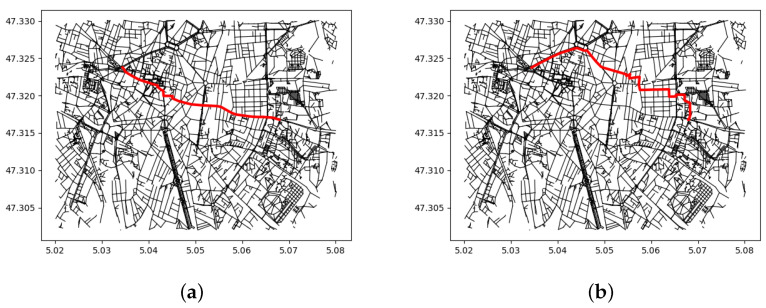
Shortest and adapted path for a blind person from a starting point to reach a desired destination: (**a**) Shortest path; (**b**) Customised navigation path for blind persons.

The A* search algorithm [42] with an Euclidean distance heuristic is chosen for its faster computation time compared to a Dijkstra search algorithm. The presence of an additional heuristic on the target geographical position helps estimate the distance from the current vertex to the target vertex, which makes it possible to eliminate unpromising paths early and focus on the most favourable paths. The resulting shortest path and customised path to reach the desired destination from a starting point are shown in Figure 4. The map file used for path-finding was filtered to remove the inaccessible areas compared to the Figure 2. The adapted path takes a longer route but prefers pedestrian streets over avenues without information about the presence of pavements. The path obtained from the path-finding method consists of a list of vertices with geographical coordinates and associated attributes, including the user’s starting position, the second-closest graph vertex, and the destination position.

**Figure 5:**
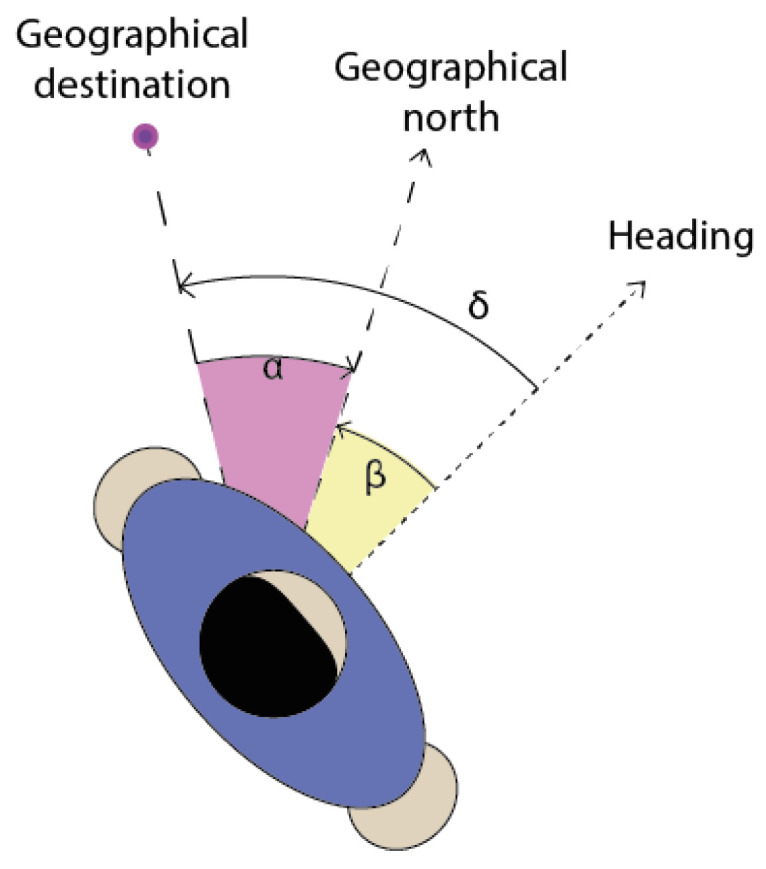
Schematic view of the determination of the angular direction according to the user orientation.

Consequently, to reach the intended destination, users must navigate through all intermediate geographical markers. These positions represent critical locations in an urban setting, such as intersections. The user’s guided path is determined by orienting them to the subsequent geographical position until the final destination is reached. This direction, called δ in Figure 5, is deduced by computing the bearing α and the heading β. The bearing indicates the directional alignment between the current position and the target position, measured clockwise from north. The bearing formula is given in Equation (1) with user and dest denoting the user’s position and destination, whilst the angular latitude θ and the longitude difference ΔL provide accurate directions.

(1) $$\begin{array}{cc} \; & X=\cos\left(\theta_{dest}\right)\times \sin\left(\Delta L\right)\\ & Y=\cos\left(\theta_{user}\right)\times \sin\left(\theta_{dest}\right)-sin\left(\theta_{user}\right)*\cos\left(\theta_{dest}\right)\times \cos\left(\Delta L\right)\\ & \alpha =\arctan(X,Y) \end{array}$$

The target position indicated by the bearing position is achieved by the user’s spatial orientation given by the magnetometer of the IMU to obtain the heading of the user compared to the magnetic north. However, the accuracy of the magnetometer is affected by the magnetic vector, which can vary with the rotation of the IMU sensor. In addition, the sensor’s location at the top of the helmet can be affected by large head movements during navigation, making it even more challenging to accurately determine the user’s heading. An approach to compensate for the variation in the magnetic vector is to use the IMU sensor’s accelerometer, particularly the gravity vector, to determine the yaw or pitch rotation of the sensor. The knowledge of the sensor rotations is used to rotate the magnetic vector to align the IMU referential with the ground floor to obtain the heading value of the compass with the Equation (2), where *M* represents the magnetic vector, ϕ and θ represent the rotation of the sensor in relative to the earth’s reference frame. The bearing information and the heading information are compared to establish the direction with an angular difference. The magnetic declination of the current location must be considered in the calculation before navigation to ensure that the heading value is centred on magnetic north and then converted to true north. However, the predicted navigation path only indicates the route to follow to reach the destination but ignores some obstacles present in an urban environment or areas of potential danger for a pedestrian person such as roads.

(2) $$\begin{array}{cc} \; & X=M_{z}*\sin\left(\phi \right)-M_{y}\times \cos\left(\phi \right)\\ \; & Y=M_{x}*\cos\left(\theta \right)+M_{y}\times \sin\left(\theta \right)\times \sin\left(\phi \right)+M_{z}\times \sin\left(\theta \right)\times \cos\left(\phi \right)\\ \; & \beta =\arctan(X,Y) \end{array}$$

### 3.2. Obstacles and Hazardous Areas Localisation

The presence of static and dynamic obstacles, combined with the presence of roads, considerably compromises the safety of blind. The white cane is adept at identifying obstacles that are low to the ground or in proximity, such as detecting the presence of a slope. However, a complementary approach is needed to identify obstacles and detect higher obstacles with their specific characteristics. A good understanding of the scene is crucial to facilitate navigation, including accurately locating nearby hazards. However, the data provided by the map may be incomplete or insufficient to detect a potential hazard. Indeed, the coordinates could be inaccurate and guide the user in the middle of the street. Consequently, the defined path must adapt to the presence of these elements to ensure safe navigation.

**Figure 6:**
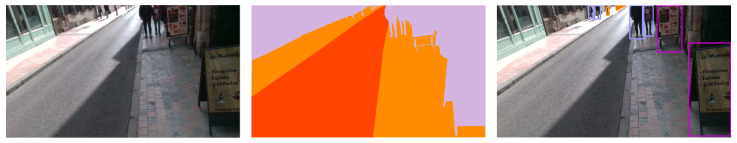
Ground segmentation (**middle**) and obstacle detection (**right**) of an RGB image (**left**).

The spatial information, captured by a helmet-mounted camera, is utilised to analyse the surrounding space around the user. The scene is analysed using segmentation and detection algorithms to determine the type of ground and information about near elements. Semantic segmentation can differentiate urban areas to identify suitable and pedestrian-friendly regions, refining predefined spatial orientation. The RGB images, as shown in the left image of Figure 6, are segmented into five essential classes: pavements, zebra crossings, roads, and background. This segmentation represents areas that are explicitly intended for pedestrians (middle image in Figure 6).

**Figure 7:**
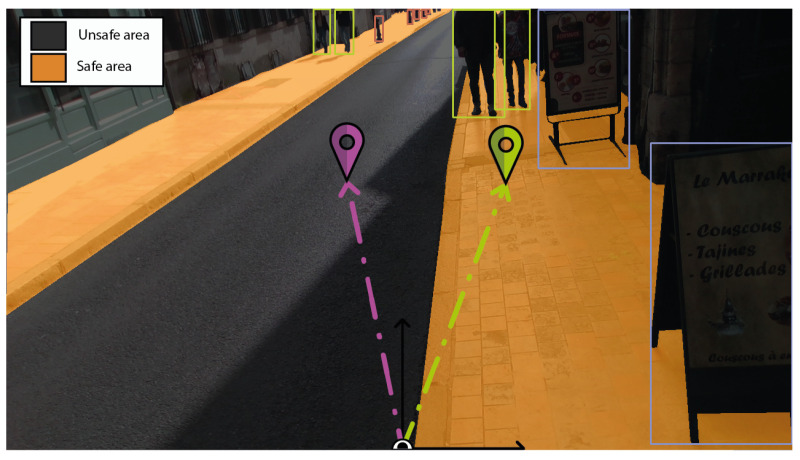
Trajectory adjustment from an initially marked position (purple icon) to a rectified location (green icon), aligning with accessible pedestrian routes.

The method for determining a path, which might initially lead to an area that is either not accessible or unsafe, is modified by pinpointing the nearest path that can be continuously and safely walked on by the user. This adjustment starts with identifying a continuous, hazard-free zone from the lower part of the segmented image. The next step involves singling out pedestrian-friendly areas, such as pavements and crosswalks, using a masking technique. This selection is further refined by excluding any zones not connected to the image’s bottom edge, indicating a break in access that necessitates navigating through a potentially dangerous area. The result of this procedure is a collection of distinct pixel clusters. Geometric analysis is then applied to these clusters to locate the nearest one. This is achieved by calculating their moments and determining the centroid of each cluster. The adjusted path is centrally located in the safer area to minimise the risk of navigating near the potentially dangerous edges of the pavement. Figure 7 illustrates the adjustment from the initial trajectory (marked with a purple icon) on the road surface to a safer route on the pavement (marked with a green icon).

On the other hand, the detection methodology discerns the presence of static or dynamic elements likely to hinder navigation, as shown in the photograph to the right of Figure 6. The detection method focuses on the localisation of 22 classes of objects, representing the most commonly observed elements in urban environments. The following list provides an overview of detected objects with an associated hazard significance, classified as low for static objects and medium to high for dynamic objects, reflecting their potential impact on navigation safety. Indeed, the level of danger of every element in the environment varies depending on its characteristics, such as nature, speed, and distance from the user. Indeed, some elements are irrelevant for the navigation and could use useless resources of the processing unit. Moreover, transmitting their position could reduce the user’s scene understanding capacity due to a cognitive overload. Consequently, prioritising the processing of relevant obstacles according to the level of danger is essential.

- Low: Tree, potted plant, light traffic (vehicle and pedestrian), pole, bollard, traffic sign, table, chair, moving sign, barricade, bench, rubbish, parking meter, bus station, and bike support.
- Medium: Person.
- High: Car, truck, bus, motorcycle, scooter, and bicycle.

**Figure 8:**
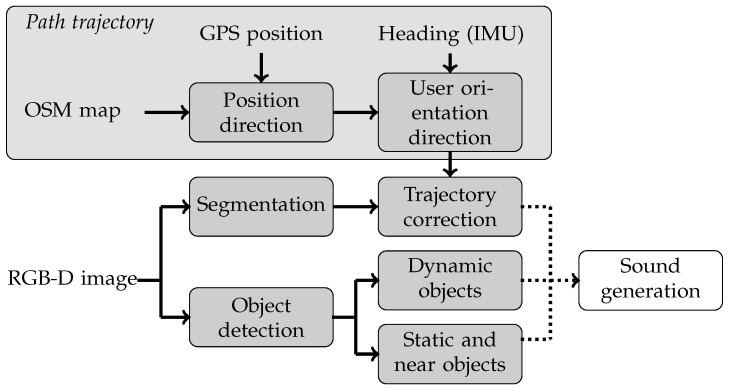
Diagram of vision processing and direction estimation.

Our method involves processing static and dynamic objects separately. Low-hazard obstacles, such as static objects or slow-moving entities like pedestrians, are processed only when they are near the user, using a depth map threshold. On the other hand, high-hazard objects in the pedestrian environment require early user action based on their movements. Dynamic objects are continuously tracked during navigation to determine whether their path poses a potential threat that necessitates avoidance or can be disregarded. The tracking method employed is the SORT algorithm [43], an acronym for simple, online and real-time tracking, summarising its feature. This algorithm combines a Hungarian algorithm [44] and a Kalman algorithm [45]. Figure 8 shows a schematic representation of the vision processing methods followed by the sound generation mechanism. This mechanism, known as sonification, merges the rectified navigation trajectory and obstacle data into global sound information.

### 3.3. Sonification

In this system, verbal phrases and short spatialised sounds are combined in order to provide relevant information to the users. Verbal phrases can provide semantic information, and we use them in special situations which, for example, occur when the user walks on a pedestrian crossing or when they reach an intermediate position, to indicate the change in orientation and the distance to the next position. These verbal phrases are emitted as monophonic sounds. However, this type of information takes too much time to indicate a direction continuously or to alert anyone of a quickly approaching obstacle, situations for which spatialised sounds are preferred. Transmitting only sounds for the direction to follow would disconnect the user from their environment, raising safety issues. On the other hand, only transmitting sounds concerning the obstacles would require further spatial processing for the brain to deduct a safe pathway between the potential dangers. For these reasons, we decided to transmit both. The sonification of obstacles is performed based on two-step processing precisely described in a previous work [46]. In the first step, the depth video stream is processed to detect the edges of objects in front of the user. In the step second step, graphical pixels considered relevant from the first step are each associated with auditory pixels. These auditory pixels are synthesised using pure tones with a frequency ranging from 250 Hz to 1492 Hz, depending on the elevation of the associated graphical pixel. The position of the associate graphical pixel in the field of view of the camera is also used to select the head-related impulse responses that are used to spatialise the auditory pixel using the CIPIC database [47]. Similarly to natural sounds, the intensity of the generated sound is directly modulated according to the obstacle’s distance. The accuracy of this encoding scheme has been evaluated in previous work, resulting in an azimuthal precision of 6.72° ± 5.82° for sonification compared to a visual condition accuracy of 2.85° ± 1.99° [29]. Additionally, after a familiarisation phase, the scheme’s elevation accuracy was recorded at 17.67° ± 22.23° [46]. The direction to follow is indicated based on the same synthesising procedure but used to transmit the azimuthal angle to the user without the distance and the elevation information. These sounds are easily distinguishable from the others due to their temporal characteristics, designing very brief sounds (50 ms) emitted every 500 ms. Finally, these two auditory streams (i.e., obstacles and direction) are merged to construct the audio output of the system.

## 4. Results

Our system transforms the rectified trajectory information and obstacle data into auditory signals to facilitate navigation for the blind. A reliable and user-friendly navigation aid must be able to perform well in different environments, provide fast and accurate signals that are easy to comprehend, and should not be affected by factors such as weight or bulkiness. Consequently, we propose the implementation of our method within an embedded system. This implementation was evaluated to determine its impact on the computation time, the robustness of deep learning approaches for obstacle and pedestrian detection, and the ability of a person equipped with our system to reach a desired destination in a series of navigation scenarios.

### 4.1. Vision Algorithm Validation

The architecture of the network and the training components, particularly the dataset used for training, play a vital role in accurately analysing a scene. Regarding the detection method, two distinct RGB image sets displaying pedestrian views were combined: SideGuide [48] and uB-Geoloc [49]. These datasets were explicitly designed to enhance visual assistance technologies’ development by annotating dynamic and static objects frequently found in pedestrian surroundings. The SideGuide dataset includes urban images from Korean cities with 29 classes commonly observed in such environments. On the other hand, UB-Geoloc consists of 16 video sequences, recorded from a European context, characterised by 28 classes. Fusing these datasets yields a variegated training dataset representative of diverse environments, thereby contributing to creating a resilient deep-learning model designed for navigation. The resulting dataset comprises the 22 classes mentioned earlier.

The selection of this network resulted from a comparison of existing deep learning architectures in the literature which could achieve both real-time inference time on an embedded system and sufficient accuracy for obstacle detection. We implemented YOLOv8 in the medium variant, known as Yolov8m, which is the detection algorithm in our system. Yolov8 is the latest version of the popular convolutional neural network (CNN) named YOLO. YOLO was specifically designed to enable real-time inference on mobile architectures while achieving high accuracy for objects of different sizes, using a single network to simultaneously predict object bounding boxes and class probabilities for those boxes. The training was performed over 100 epochs with an initial learning rate of 0.1, using 640×640 pixel resolution scaled images to achieve a high detection accuracy. The model exhibited high precision on the test dataset, with a clear distinction between different classes, accurately classifying 168,888 out of 170,249 obstacles and resulting in a correct classification rate of 99.2%. Moreover, the model achieved an average recall of 78% and an average precision of 86.9%, alongside a mean average precision (mAP) at a 50% intersection over union (IoU) of 85.9% with detailed class-wise results presented in Table 1. The model’s low confusion score significantly reduces the risk of misjudging the potential danger posed by obstacles during navigation. The main sources of error in the prediction and recall rates are difficulties in detecting distant objects or incorrect background recognition. For example, the system may fail to recognise a person as an obstacle at a significant distance. However, these distant elements are less relevant than a nearby element for pedestrian navigation. Furthermore, these detection errors decrease as the distance between the user and the object decreases, and the object becomes increasingly relevant to the user. The use of depth data in our method improves the detection robustness by eliminating irrelevant distant obstacles using the depth value provided by the RGB-D camera and used in the sound encoding scheme.

On the other hand, the segmentation network was trained using a combination of the SideGuide semantic annotation set and the Mapillary dataset. The fusion of these two relevant datasets enables our segmentation method to be widely used within many urban spaces and camera orientations. The annotation part of the SideGuide dataset provides annotations of pedestrian images in an urban setting, with the camera focused on capturing the first floor and excluding background information. In contrast, Mapillary [50] includes a wide range of annotated images incorporating background details. However, the perspective is often from the driver’s seat in a vehicle rather than a pedestrian viewpoint.

**Figure 9:**
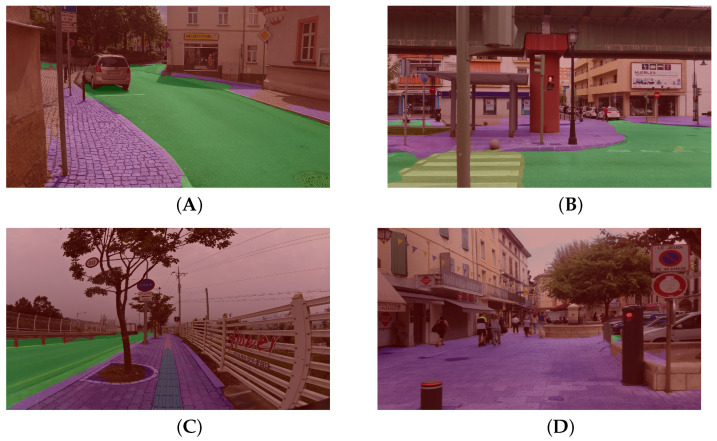
Examples of accessible path prediction for a severely visually impaired person. The colours green (road) and red (background) represent dangerous paths. Safe paths are indicated by the colours purple (pavement), yellow (pedestrian crossing), and grey (tactile walking surfaces). (**A**). Cobblestone pavement; (**B**). Intersection; (**C**). Blind pavement; and (**D**). Pedestrian area.

The CNN architecture implemented is a DDRNET, selected using the same criteria as the detection model. This model has trained over 120,000 iterations with a batch size of 24 images, using input images with a resolution of 1024×512 pixels. The results of evaluating the trained model and using the standard metrics of the segmentation task are presented in Table 2. The network demonstrates remarkable accuracy in segmenting the urban area, skilfully differentiating different parts of the image according to their corresponding classes. This is evidenced by several examples taken from the test dataset, as illustrated in Figure 9. However, semantic segmentation processing can be hindered by certain environmental conditions, such as snow-covered roads or fog, due to a lack of diverse dataset samples representing these scenarios. A training dataset enhanced with varied weather conditions of the visual information could limit this issue. Additionally, the limited resolution of the input image compared to the source image notably influences the accuracy with which mislabelled regions mainly manifest along the edges of the segmentation blobs (Figure 9A,B) or thin and distant area (Figure 9C), but has no impact on our method in which we search a large area to navigate (Figure 9D). In addition, erroneous labels are eliminated by a filtering process that integrates a morphological operation, as well as the thresholding of the probability of each pixel. In this way, the trajectory can be rectified to ensure safe navigation for a severely visually impaired user, as described earlier in the method section.

### 4.2. System Performance

**Figure 10:**
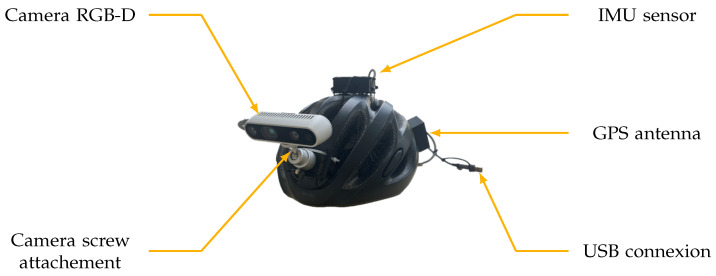
Photography of the acquisition system.

The overall method was implemented to propose a realist system to assist a blind person in real time during a navigation task to reach a desired destination. The device was composed of a helmet with several components, represented in Figure 10, including an Intel Realsense D435 RGB-D camera, an Adafruit BNO055 IMU sensor, and an Adafruit Ultimate GPS breakout with an acquisition’s frequency, respectively, of 30 frames per second, 100 Hz and 10 Hz. The helmet system was connected to a processing unit in the user’s backpack using a USB wire. The same connection also functioned as a power supply. The processing unit used for this implementation was a laptop on Ubuntu 20.04 equipped with an Intel Core I7 12700h processor, which had 14 cores/20 threads, 16 GB of RAM, an RTX 4050 mobile graphic board, and a 90W/h battery. The program was written in C++ using OpenCV, Eigen and Librealsense-2 libraries.

**Figure 11:**
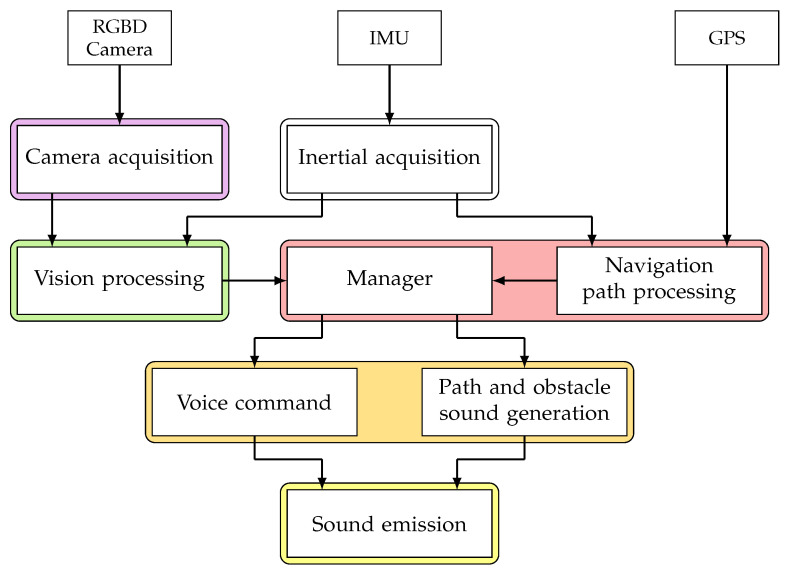
Diagram of the processing chain, from data acquisition to sound emission, where each wire corresponds to a single colour.

The latency from the data acquisition to the sound emission of the orientation or potential hazard is a crucial part of assistive technology. Extended delays between successive updates can lead to discomfort during navigation, as the provided guidance may no longer align with the user’s real-time position in the environment. We propose a multithreaded architecture approach to reduce the delay illustrated in Figure 11. This architecture comprises five concurrent threads devoted to specific information acquisition and processing tasks. The *Manager* thread orchestrates the global processing of user navigation orientation and auditory scenarios. This includes the emission of spatialised sounds related to proximate elements and providing verbal commands when necessary, such as querying the user’s intended destination. Additional threads segment the overall process into tasks following the traditional SSD pipeline. Specifically, two threads focus on data acquisition, encompassing IMU and RGB-D images, while two other threads handle visual processing for detection and semantic segmentation. The final two threads are dedicated to sound generation and emission. Including a vast navigation space with many vertices and edges is indispensable for crafting a robust pedestrian navigation method capable of efficiently executing navigation algorithms in dense or vast environments without a significant time computation. The navigation thread uses inertial data to obtain compass orientation, and the vision processing thread to correct small camera movements made by users.

The overall processing time of the system is heavily influenced by the cumulative processing times of its individual stages. The precise measurement of each stage’s processing time is reported in Table 3 and Table 4. The camera was bypassed during the processing time measurement to limit the influence of frame rate delays on the overall processing time. The system enables real-time execution thanks to GPU acceleration without requiring the total capacity of the laptop. The CPU utilisation peaked at 13.70%, accompanied by a RAM usage of 4.7 GB. GPU utilisation reached a maximum of 76%, with VRAM utilisation of 1.337 GB and power consumption of 74 watts. However, the CPU implementation of the method encounters difficulties in satisfying computational loads, leading to slower and less responsive processing. Consequently, the system configuration cannot guarantee comfortable, fluid, and safe navigation, which underlines the crucial importance of GPU acceleration in achieving the optimal system performance. We used a filtered map of the Burgundy region in France during the experimentation, directly extracted from the OpenStreetMap website with 95,918 vertices and 801,116 edges.

We propose implementing our method on an embedded target, aiming to maximise the system’s battery life and ensure an extended navigation time while reducing the occurrence of system power-down during navigation, which can lead to feelings of insecurity. The decision to integrate an embedded target instead of a laptop is driven by the low hardware resource cost of our method on the laptop, allowing for the selection of a lower-capacity hardware platform without significantly compromising results, thereby reducing the power consumption. The embedded implementation was executed on a Nvidia Orin AGX 32 GB, a robust ARM/GPU System-on-Chip (SoC). The primary optimisation goal was to manipulate the deep learning model’s power consumption and floating-point characteristics to enhance its compatibility with prior optimisations. However, it should be noted that hardware platforms such as ARM/FPGA SoCs [51] also present interesting performance possibilities when combined with techniques for the efficient implementation of deep learning networks. The modification of the floating point from 32 bits to 16 bits does not impact the accuracy of the network, as a mixed-precision configuration was adopted during training. Table 5 provides a comparative summary of processing times across the different configurations of the power mode and floating point precision. A power mode is a predefined setting by the chip manufacturer that specifies the frequency and number of cores activated to limit power consumption. The resulting system enables real-time processing with low latency, allowing outdoor experiments to be proposed.

### 4.3. Experimentation

We measured the effectiveness of a navigational aid designed to assist by emitting short sound cues that convey directional guidance and obstacle warnings within an urban context. A blindfolded participant was selected to test the system to replicate the visual impairment conditions. After learning the sound codes used by our aid, the participant was asked to find their way to an undisclosed destination, using only the audio cues from our system. A sighted guide was present during the test to offer protection against potential hazards or misinterpretations of audio signals and to aid in safe road crossings. The experiment was controlled by a supervisor who programmed the destination into the system without revealing it to the user. Different navigation scenarios were performed, such as moving through large pedestrian areas and along city streets, to test the system and human behaviour. The participant’s movement was tracked by the GPS antenna and displayed on a 2D map.

**Figure 12:**
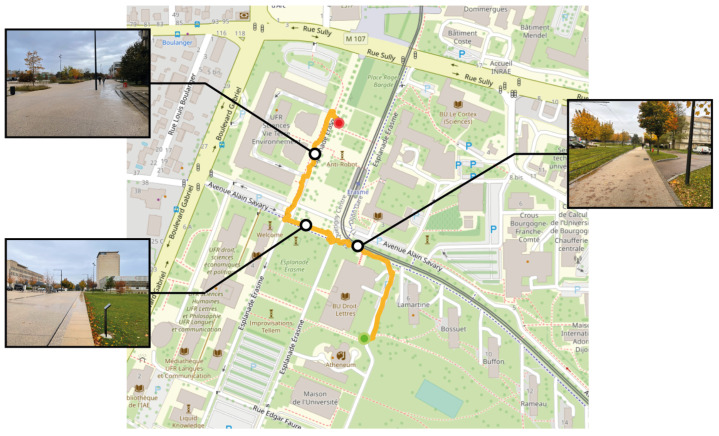
Map view illustrating a pedestrian’s way to a destination, with a red dot marking the start and a green circle marking the end. Black dots highlight significant landmarks along the way, each paired with a visual snapshot: the top-left shows a vast navigation area, the bottom-left depicts a space with a pronounced step from the pedestrian way to the grassy area, and the left-most view captures an intersection.

The initial experiment took place on a university campus chosen explicitly for its controlled environment, which made it ideal for evaluating human–machine interaction in real-world scenarios. Despite its safety, the campus setting introduced realistic elements to the navigation task by including pedestrian traffic, which increased the complexity of the test. The participants were required to navigate various pathways and intersections to locate a designated building on the campus. The map shown in Figure 12 provides a detailed visual account of the participant’s route, encompassing diverse elements of the campus landscape, such as an open esplanade and an intersection. The participant began at a red-marked location, initially heading in the opposite direction to reach the nearest node of the path. After reaching the nearest point, they changed direction to follow the predefined path, using auditory cues to reach the destination. Furthermore, the participant avoided obstacles and dangerous areas, such as the critical step between the footpath and the grassy area. The experiment path had a length of 385 m and was completed in 12 min, equating to an average walking speed of approximately 2 km per hour.

**Figure 13:**
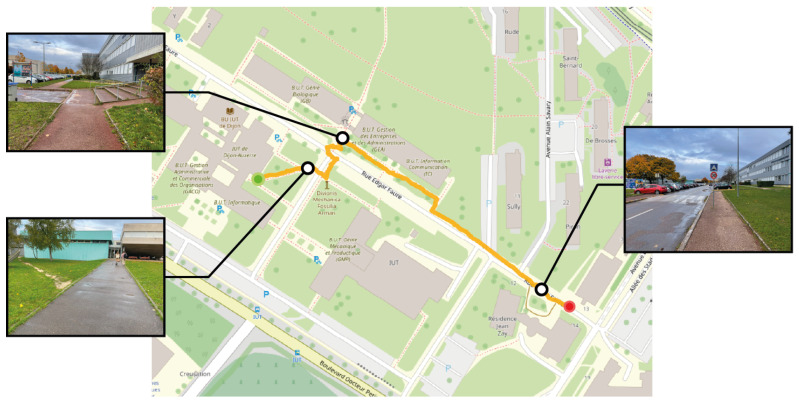
Map view illustrating a pedestrian’s route to a destination, with a red dot marking the start and a green dot marking the destination. Black dots highlight significant landmarks along the way, each paired with a visual snapshot: the top views display the area traversed on the pavement, and the bottom left shows a narrow trail used by the pedestrian.

The second navigation test was in a hybrid environment characterised by the intersection of pedestrian walkways and motor vehicle routes. Similar to the first experimentation, the participant was tasked with reaching a predetermined destination, with their route illustrated in Figure 13. This figure highlights the critical features of the street layout, including obstacles like poles and the pavement’s edge, providing context for the navigational challenges encountered. The navigation algorithm prioritised pedestrian-friendly service paths due to their lower associated travel costs than secondary roads. This preference led the participant away from the pavement and onto a footpath running alongside a building, before later rejoining the main road. The figure also features a detour to a less prominent dirt path (bottom left visual snapshot). This path, although less prominent, was present in the pre-established navigation path and was accurately identified by the segmentation algorithm as a potential route. The navigation along this path covered approximately 290 m and was completed in about 10 min, corresponding to an average pace of 1.7 km per hour.

**Figure 14:**
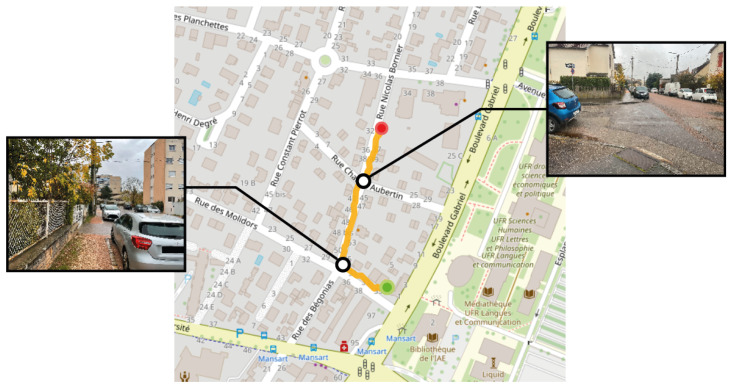
Map depicting a pedestrian’s pathway through an urban environment, marked by a red dot at the origin and a green dot at the destination. A black dot showcases the navigation zone, particularly the pavement, accompanied by the visual perspective of the area.

Finally, the user had to navigate to a specific location in a moderately dense urban environment, characterised by narrow pavements, free-flowing traffic, and vehicles parked along the streets, corresponding to a standard urban neighbourhood. The location was similar to a typical neighbourhood in a city. The user was able to cover a distance of 232 m in 8 min with no navigational issues, except at an intersection where supervisory assistance was required for crossing. The lack of marked pedestrian crossings impeded the participant’s ability to traverse the road independently. Consequently, the supervisor helped the user to cross the road once the absence of obstacles had been audibly perceived by the user. Nevertheless, the blindfolded individual skilfully moved between the pavement and the parked cars during the exercise. The route followed by the individual is illustrated in Figure 14, and the problematic intersection is indicated by the black dot on the right.

In the experimental part, we evaluated the blindfolded user’s ability to navigate in the urban environment using a combination of two types of audio information representing the path and obstacles. The user demonstrated their ability to navigate in an unfamiliar space without encountering any obstacles, despite some apprehension due to the lack of usual visual information during navigation. However, the combination of the two auditory cues gave sufficient confidence to perceive the surrounding information.

## 5. Conclusions

We introduced an innovative outdoor navigation system designed for the blind. This system provides a means to navigate urban environments using 3D spatial audio cues. This advanced system combines environmental data and trajectory information based on visual and spatial inputs to generate immediate low-latency audio signals. These signals inform users of imminent obstacles and dynamic elements in their path, enabling their safe and efficient navigation with deep-learning approaches. We conducted a comprehensive performance evaluations of the system, as well as user behaviour studies, to assess its practical application. Indeed, the accuracy of the visual processing approaches has been evaluated to ensure high robustness in different urban environments. In addition, the proposed system was evaluated with performance measurements indicating real-time processing on an embedded board with moderate power consumption. Finally, our system was also subject to qualitative validation in outdoor environments. Various urban navigation scenarios were conducted using a subject wearing a blindfold to evaluate the system’s effectiveness and to study human responses to our encoding scheme. These evaluations revealed some limitations, with a prominent concern being the potential degradation of GPS signals in urban landscapes. The presence of tall buildings may impede signal accuracy, which is a significant consideration. However, we found that incorporating visual data significantly enhances reliability, offsetting GPS limitations. The system currently does not provide explicit guidance for navigating unmarked pedestrian crossings or degraded weather conditions such as fog or snow on the ground, which are a limitation in large-scale urban mobility. Additionally, while it can detect pedestrian traffic lights, a more sophisticated encoding method is needed to fully support complex pedestrian navigation scenarios. Despite these challenges, our research demonstrates that the system associated with a white cane substantially supports severely visually impaired users in their quest to independently reach desired destinations in an urban setting. This solution is a complementary aid, supplementing the invaluable support typically offered by human assistance when such help is unavailable. The positive outcomes suggest that, with further refinement, this system could become an indispensable tool for enhancing the autonomy and safety of the blind in navigating urban environments.

## Figures and Tables

**Table 1:** Average precision at a 50% intersection over union (IoU) threshold for the obstacle detection.

Car	Truck	Bus	Motorcycle	Scooter	Bicycle
96%	92.3%	88.8%	88.8%	76.4%	89.6%
Person	Tree trunk	Potted Plant	Traffic light	Pole	Bollard
87.7%	86.3%	82.2%	84.6%	87.2%	83.6%
Traffic sign	Table	Chair	Moving sign	Barricade	Bench
84.6%	86.9%	85.7%	82.6%	81.9%	72%
	Rubbish	Parking meter	Bus station	Bike support	
	83.2%	90.6%	90.4%	91.5%	

**Table 2:** Accuracy of the pedestrian segmentation model evaluated on a fusion of the Mapillary and SideGuide datasets.

Pixel Acc.	mAP	Background	Road	Sidewalk	Crosswalk	Tactile Pav.
92.299%	85.61%	86.17%	87.37%	81.22%	86.24%	87.06%

**Table 3:** Processing time comparison for sound processing and trajectory estimation computation.

Path Estimation and Correction	Sound Generation	
	Path Trajectory	Obstacle
2 ms	<0.1 ms	0.4 ms

**Table 4:** Comparison of vision processing times and overall processing times for a CPU and GPU implementation.

	Vision Processing		Global Processing
	Segmentation	Detection	
CPU—Libtorch (ms)	1130	142	1136
GPU—TensorRT (ms)	4.9	10.4	14.2

**Table 5:** Comparison of processing time for vision and global processes with different power consumption modes and floating point dynamics.

	FP32 MaxN	FP32 50 W	FP32 30 W	FP16 MaxN	FP16 50 W	FP16 30 W
Detection (ms)	17	23	46	8	12	26
Segmentation (ms)	9	14	36	4	6	18
Global processing (ms)	27	38	81	20	27	44

## Data Availability

Data are contained within the article.
